# The Impact of Normal Range of Serum Phosphorus on the Incidence of End-Stage Renal Disease by A Propensity Score Analysis

**DOI:** 10.1371/journal.pone.0154469

**Published:** 2016-04-28

**Authors:** Wen Xiu Chang, Ning Xu, Takanori Kumagai, Takeshi Shiraishi, Takahiro Kikuyama, Hiroki Omizo, Kazuhiro Sakai, Shigeyuki Arai, Yoshifuru Tamura, Tatsuru Ota, Shigeru Shibata, Yoshihide Fujigaki, Zhong Yang Shen, Shunya Uchida

**Affiliations:** 1 Department of Nephrology, Tianjin First Central Hospital, Tianjin, China; 2 Support for Community Medicine Endowed Chair, Teikyo University School of Medicine, Tokyo, Japan; 3 Department of Internal Medicine, Teikyo University School of Medicine, Tokyo, Japan; 4 Department of Organ Transplantation, Tianjin First Central Hospital, Tianjin, China; University of São Paulo School of Medicine, BRAZIL

## Abstract

**Background:**

Although hyperphosphatemia is deemed a risk factor of the progression of chronic kidney disease (CKD), it remains unclear whether the normal range of serum phosphorus likewise deteriorates CKD. A propensity score analysis was applied to examine the causal effect of the normal range of serum phosphorus on the incidence of end-stage renal disease (ESRD).

**Methods:**

A retrospective CKD cohort of 803 participants in a single institution was analyzed. Propensity score was estimated using 22 baseline covariates by multivariate binary logistic regression for the different thresholds of time-averaged phosphorus (TA-P) in the normal range of serum phosphorus incremented by 0.1 mg/dL from 3.3 to 4.5 mg/dL.

**Results:**

The incidence rate of ESRD was 33.9 per 1,000 person-years over median follow-up of 4.3 years. Total patients showed the mean baseline phosphorus of 3.37 mg/dL and were divided to quartile. The higher quartile was associated with the parameters consistent with the advancement of CKD. A stratified Cox regression showed the highest hazard ratio (HR) at TA-P 3.4 mg/dL (HR 17.60, 95% CI 3.92–78.98) adjusted for baseline covariates such as sex, age, diabetic nephropathy, estimated GFR, serum albumin, Na-Cl, phosphorus, LDL-C and proteinuria. Adjusted HRs remained high up to TA-P 4.2 mg/dL (HR 2.22, 95% CI 1.33–3.71). After propensity score matching conducted at the thresholds of TA-P 3.4, 3.6, 3.8 and 4.0 mg/dL, the higher levels of TA-P showed the higher HRs by Kaplan-Meier analysis (p < 0.05 by stratified log-rank test). The numbers needed to treat were calculated as 3.9 to 5.3 over 5 years.

**Conclusions:**

The propensity score analysis shows that even the normal range of serum phosphorus clearly accelerates CKD progression to ESRD. Our results encourage clinicians to target serum phosphorus to inhibit CKD progression in the manner of ‘the lower the better.’

## Introduction

Early recognition and intervention against risk factors responsible for the progression of chronic kidney disease (CKD) are expected to improve renal outcomes of patients at risk [1]. Major risk factors of subsequent incidence of end-stage renal disease (ESRD) are anemia, proteinuria and hypertension in addition to preceding kidney dysfunction [2, 3]. However, the second line of modifiable risk predictors remain to be clarified; candidates are hypoalbuminemia, hyperuricemia, hyperphosphatemia, metabolic acidosis, dyslipidemia, etc.

Most recently we have found that the higher normal range of serum phosphorus, either at baseline or in the follow-up, may be a strong risk factor of CKD progression towards ESRD [4, 5]. A rapid progression group of CKD patients defined by 25% decline in estimated GFR per year was associated with serum phosphorus with the highest odds ratio (OR 6.5, 95% CI 2.8–14.9) comparable to that of proteinuria. The cut-off value of phosphorus in the follow-up was estimated at 3.82 mg/dL [4]. Using the dataset having two measurements of serum creatinine 2-year apart, we further examined the significance of 30% decline in estimated GFR over 2 years as a novel surrogate endpoint for the CKD progression [6]. During this investigation we found that serum phosphorus over 2 years had strong influence on the future incidence of ESRD (HR 2.70, 95% CI 1.54–4.76) [5]. Moreover, 30% decline in estimated GFR over 2 years was associated with proteinuria, hemoglobin, uric acid, phosphorus and male. Again the cut-off value of serum phosphorus over 2 years was as low as 3.51 mg/dL [5]. These results prompted us to speculate that serum phosphorus, even within the normal range, may accelerate the progression of CKD if estimated GFR less than 60 mL/min/1.73 m^2^.

To explore this hypothesis, we conducted a standard survival analysis using serum phosphorus in the follow-up and a propensity score-based survival analysis, the latter of which is increasingly being used to estimate causal effects in the observational studies because one can replicate the prospective randomized controlled trial by minimizing baseline confounding [7]. We utilized two propensity score methods; the stratified Cox proportional hazards model and the matching method followed by the Kaplan-Meier analysis according to our recent study [8].

## Patients and Methods

### Study protocol and ethical statement

We used a retrospective CKD cohort already reported (n = 803) [4, 5, 8], and in the current study we repeated the propensity score-based stratified Cox regression methods and the propensity score matching that we have used in our previously published article [8]. Inclusion criteria consisted of CKD stage 3 to 4, age 20 to 84 years and follow-up period ≥ 1 year. On the other hand, patients with nephrotic syndrome, malignancy, obstructive nephropathy, acute kidney injury and gout were excluded. All the patients were followed up to 6 years until censoring or reaching the initiation of dialysis. The present study was approved by the institutional review board (IRB) in the Teikyo University Review Board #14–115 and was executed in accordance with the principle of the Helsinki Declaration. Written informed consent was waived after approval of IRB and the patient records and information was anonymized and de-identified prior to analysis.

### Parameters analyzed

The demographic characteristics included sex, age, body mass index (BMI), original kidney disease (diabetic nephropathy or not) and systolic blood pressure (SBP). Blood parameters included hemoglobin (Hb), white blood cell (WBC), platelet (Plt), albumin (Alb), uric acid (UA), sodium (Na), potassium (K), chloride (Cl), Na-Cl (as a surrogate of HCO_3_), albumin-corrected calcium (cCa), inorganic phosphorus (P), low-density lipoprotein cholesterol (LDL-C) and C-reactive protein (CRP). Urine parameters included spot urine proteinuria (expressed as gram per gram creatinine excretion) and spot urine hematuria by dipstick (coded as four grades of 0 to 3 according to 0, 1+, 2+, and 3+ and as 0.5 if ±). Due to the distribution, C-reactive protein, proteinuria and hematuria were log-transformed for analyses.

Blood was tested using hematology autoanalyzer (Sysmex XE-5000, Kobe, Japan) and blood chemistry parameters were measured by routine measurements using autoanalyzer (LABOSPECT 008, Hitachi High-Technologies Corporation, Tokyo, Japan). Creatinine concentration in serum and urine was measured by an enzymatic method. Serum phosphorus was measured by an enzymatic method with malachite green and urinary protein concentration measured by a pyrocatechol violet-metal complex assay method. Serum phosphorus measured at every visit was recorded until censoring or reaching estimated GFR 5 mL/min/1.73 m^2^ and calculated as time-averaged phosphorus (TA-P) by trapezoidal rule [8]. Estimated GFR was evaluated using the Modification of Diet in Renal Disease (MDRD) study equation for Japanese population [9]. The grade of CKD was classified based on the Kidney Disease Outcomes Quality Initiative (K/DOQI) practice guidelines [1].

Use of antihypertensives including angiotensin converting enzyme inhibitor or angiotensin II receptor blocker (combined as RASi), use of diuretics and use of phosphate binders were recorded as yes (coded as 1) or no (coded as 0). Only four patients received phosphate binder (all carbonic calcium) at entry thus the information was not used any further. The baseline covariates used for the propensity score estimate modeling became 22 in total.

### Endpoints of renal outcomes

A primary endpoint was defined as the incidence of ESRD (initiation of hemodialysis or peritoneal dialysis). Death was treated as censoring because the present study focused on the effect of phosphorus on the subsequent ESRD rather than the risk of mortality [5, 10]. Competing risk method was not employed due to two reasons; death censoring was found only 10 patients [11] and inability of performing this method in the propensity score analysis.

### Standard Cox regression analysis using time-averaged phosphorus in the follow-up

The effects of higher levels of TA-P by dividing to two groups on the incidence of ESRD were examined using a standard Cox proportional hazards model. Threshold was incremented by 0.1 mg/dL of TA-P from 3.3 to 4.5 mg/dL. Following the univariate analysis of TA-P for the incidence of ESRD, a multivariate analysis was adjusted for demographic parameters such as sex, age, diabetic nephropathy and baseline covariates including estimated GFR, albumin, Na-Cl, phosphorus, LDL-C and proteinuria as previously [8].

### A propensity score analysis

The threshold of TA-P in the follow-up was tested from 3.3 to 4.5 mg/dL by an increment of 0.1 mg/dL. The probability to reach above or equal to the threshold was determined by a multivariate binary logistic regression using the aforementioned 22 baseline covariates. Since the distribution of propensity score of two groups differed widely, patients whose propensity scores not overlapped between two groups were trimmed, then the remaining subsample was re-stratified on the quintiles of the propensity scores [12, 13].

#### Stratification of Cox proportional hazards model

A stratified Cox proportional hazards model was conducted in the substrata on the quintiles of the propensity scores [7]. Then, a pooled hazard ratio (HR) of the higher group of TA-P was obtained as a crude HR. Survival analysis was similarly adjusted for the nine baseline covariates.

#### Matching followed by Kaplan-Meier method

Participants divided by the designated threshold of TA-P (3.4, 3.6, 3.8 or 4.0 mg/dL) were matched using a greedy method with a 1:1 pair. The caliper size was set at 0.20 times standard deviation of the logit of the propensity scores [7]. The model of assignment was estimated by c-statistics, and the balance between two groups was checked by paired comparison tests and standardized differences of the 22 baseline covariates [14]. A survival analysis was examined by the Kaplan-Meier method with stratified log-rank test [7, 14]. Moreover, HR, absolute risk reduction (ARR) and number needed to treat (NNT) were computed [8, 15–17].

### Statistical analyses

Values for categorical variables are given as number (percentage) and values for continuous variables are given as mean ± standard deviation or median [interquartile range] depending on the distribution. The relationship between baseline phosphorus and TA-P were tested by Pearson’s correlation analysis. C-statistics for the accuracy of the propensity score models were obtained by the area under the receiver operating characteristic (AUROC) curve for the threshold [18]. Hosmer-Lemeshow test was used for estimating the goodness-of-fit of the propensity score model. Difference between two groups was examined by unpaired *t* test and chi-squared test before matching while the data after matching were compared by paired *t* test and McNemar test or Cochran Q test as appropriate [19]. Standardized differences between two groups before and after matching were calculated for each covariate and a value < 0.1 was regarded as supporting the balance between the groups [18, 20]. For a Cox proportional hazards model, any covariate was tested for its proportional hazards assumption using both a time-dependent Cox regression and a Schoenfeld residual plot. Goodness-of-fit of the proposed model was measured by Akaike information criterion (AIC) [21]. Statistical analyses were performed using SPSS version 22 (IBM, Tokyo) and STATA version 14 (StataCorp LP, College Station, TX, USA). A *p* value less than 0.05 was considered statistically significant.

## Results

### Clinical Characteristics of the Cohort

**Fig 1:**
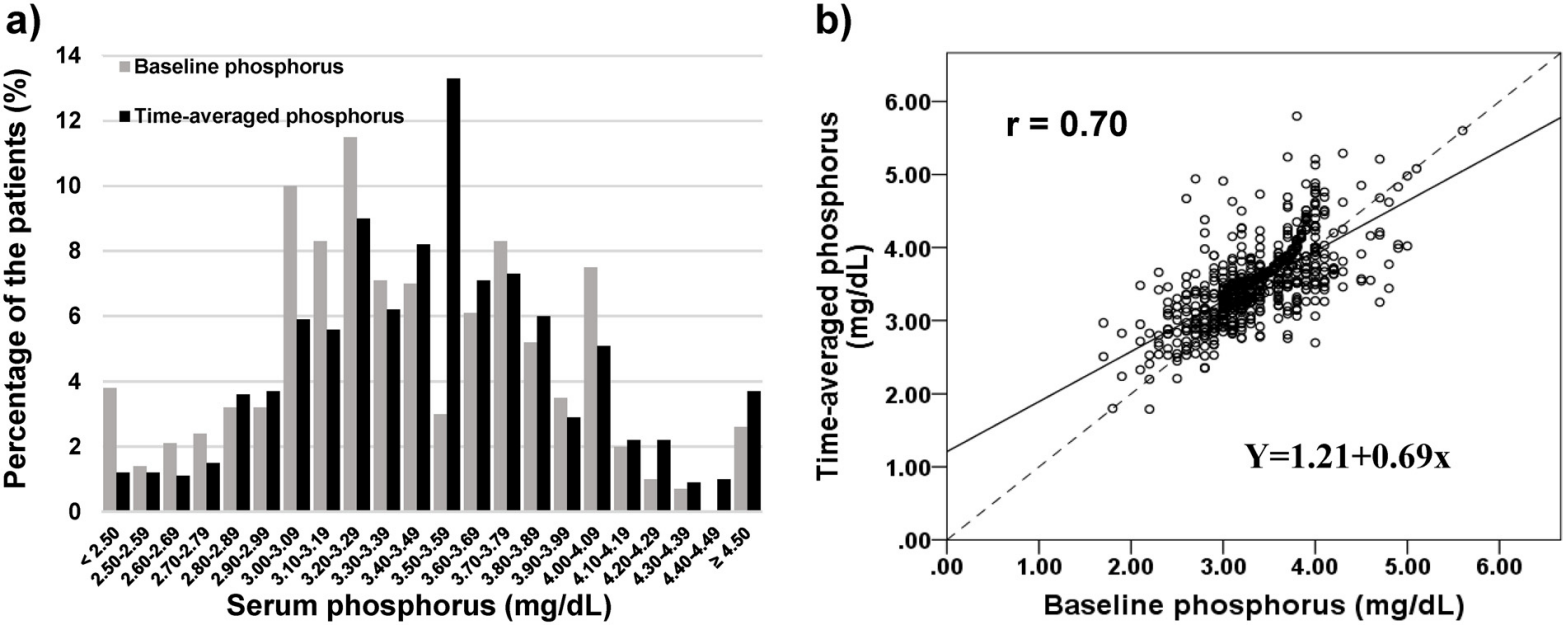
Baseline phosphorus and time-averaged phosphorus in the follow-up. **a)** The distribution of the baseline phosphorus and TA-P. The number of patients decreased below 3.5 mg/dL of serum phosphorus while the percentage dramatically increased in the range of 3.50–3.59 mg/dL of time-average phosphorus. Hyperphosphatemia accounted for 2.6% and 3.7% at baseline and in the follow-up, respectively. **b)** The relationship between TA-P and baseline phosphorus. A regression line is depicted in black line and a unity of the slope is overlapped in a dotted line. The correlation coefficient was 0.69 (*p* < 0.001). Two lines converge at 3.9 mg/dL of phosphorus. TA-P increased below baseline phosphorus 3.5 mg/dL and decreased above baseline phosphorus 3.5 mg/dL due to the remarked increase in distribution as shown in **a)**. Abbreviation: TA-P, time-averaged phosphorus.

During the follow-up period of median 4.3 [interquartile range 2.6–5.7] years, 110 out of 803 patients progressed to ESRD. The incidence rate was 33.9 per 1,000 person-years. The mean baseline phosphorus was 3.37 ± 0.52 mg/dL. The distribution of baseline phosphorus was almost normal as depicted in Fig 1a. Hyperphosphatemia defined as > 4.5 mg/dL occupied only 2.6%. The baseline characteristics were divided to quartile; < 3.05, 3.05–3.29, 3.30–3.74 and ≥ 3.75 mg/dL. But the number of the patients in each quartile was not the same due to many ties (Table 1). The differences among four groups were observed in estimated GFR, sex, CKD stages, diabetic nephropathy, hemoglobin, albumin, uric acid, potassium, Na-Cl and proteinuria. Briefly the patients having higher baseline phosphorus was associated with clinical parameters relating to the advancement of kidney dysfunction. Of note is that female preponderance was seen in the quartiles of higher baseline phosphorus (Table 1).

**Table 1:** Clinical characteristics of the cohort (n = 803).

Characteristics	Quartile 1	Quartile 2	Quartile 3	Quartile 4	*p* value*
	< 3.05	3.05–3.29	3.30–3.74	≥ 3.75	
Numbers	199	170	220	214	
TA-P (mg/dL)	3.1±0.4	3.4±0.3	3.6±0.3	4.0±0.5	< 0.001
Age (y)	62.6±13.5	62.2±12.6	62.2±12.6	61.7±13.5	0.9
Baseline eGFR (mL/min/1.73 m^2^)	43.2±12.2	43.7±12.2	41.1±13.6	37.1±13.5	< 0.001
Sex					< 0.001
Male (%)	162 (81.4)	103 (60.6)	125 (56.8)	111 (51.9)	
Female (%)	37 (18.6)	67 (39.4)	95 (43.2)	103 (48.1)	
CKD stage					< 0.001
Stage 3a (%)	98 (49.2)	92 (54.1)	99 (45.0)	75 (35.0)	
Stage 3b (%)	65 (32.7)	52 (30.6)	63 (28.6)	63 (29.4)	
Stage 4 (%)	36 (18.1)	26 (15.3)	58 (26.4)	76 (35.5)	
DMN (%)	24 (12.1)	33 (19.4)	65 (29.5)	64 (29.9)	< 0.001
BMI (kg/m^2^)	24.4±4.3	24.2±4.5	24.2±4.3	24.5±4.3	0.8
SBP (mmHg)	136.8±21.7	134.3±19.1	137.4±19.9	140.1±22.3	0.06
Blood Parameters					
Hb (g/dL)	13.5±1.9	13.1±1.8	12.7±1.8	12.1±1.9	< 0.001
WBC (×10^2^/μL)	67.1±23.8	64.5±21.1	65.8±20.9	64.5±19.9	0.6
Plt (×10^4^/μL)	21.7±6.8	21.0±6.6	22.5±6.9	22.5±6.8	0.09
Alb (g/dL)	4.0±0.5	4.1±0.4	4.0±0.5	4.0±0.5	< 0.001
UA (mg/dL)	6.5±1.3	6.1±1.4	6.5±1.4	6.7±1.5	0.004
Na (mEq/L)	140.9±2.7	140.9±2.6	140.5±3.0	140.7±2.3	0.6
K (mEq/L)	4.3±0.4	4.4±0.5	4.5±0.5	4.6±0.6	< 0.001
Na-Cl (mEq/L)	35.6±2.1	35.8±2.5	35.5±2.5	34.8±2.7	< 0.001
cCa (mg/dL)	8.8±0.4	8.8±0.4	8.9±0.5	8.9±0.5	0.5
P (mg/dL)	2.7±0.3	3.2±0.1	3.5±0.3	4.0±0.3	< 0.001
CRP (mg/dL)	0.09 [0.05–0.22]	0.09 [0.05–0.18]	0.08 [0.05–0.20]	0.07 [0.03–0.20]	0.7
LDL-C (mg/dL)	111.0±30.7	115.0±29.0	110.2±30.8	108.2±31.0	0.2
Urine Parameters (spot)					
TPU/CrU (g/g Cr)	0.33 [0.17–0.86]	0.33 [0.15–0.92]	0.48 [0.18–1.15]	0.62 [0.20–1.85]	< 0.001
UB_score	0.00 [0.00–0.50]	0.00 [0.00–0.50]	0.00 [0.00–0.50]	0.00 [0.00–1.00]	0.4
Drug use					
RASi (%)	107 (53.8)	91 (53.5)	119 (54.1)	120 (56.1)	0.9
Diuretic (%)	21 (10.6)	29 (17.1)	36 (16.4)	42 (19.6)	0.08
Outcome					
SRD (%)	13 (6.5)	14 (8.2)	30 (13.6)	53 (24.8)	< 0.001

Note: Values for categorical variables are given as number (percentage); values for continuous variables are given as mean ± standard deviation or median [interquartile range]. For statistical analyses, CRP, TPU/CrU, UB_score were log-transformed. Conversion factors for units: creatinine in mg/dL to μmol/L, x 88.4; uric acid in mg/dL to μmol/L, x 59.48. Of note is that the number of each quantile is not the same due to many ties.

* ANOVA or chi square test as appropriate.

Abbreviations: TA-P, time-averaged phosphorus; eGFR, estimated glomerular filtration rate; DMN, diabetic nephropathy; BMI, Body mass index; SBP, systolic blood pressure; Hb, hemoglobin; WBC, white blood cell; Plt, platelet; Alb, albumin; UA, uric acid; Na, sodium; K, potassium; Cl, chloride; cCa, albumin-corrected calcium; P, phosphorus; CRP, C reactive protein; LDL-C, low-density lipoprotein cholesterol; TPU/CrU, urine total protein divided by urine creatinine; UB_score, urine blood score; RASi, RAS inhibitor; ESRD, end-stage renal disease.

Next, the distribution of the TA-P was examined and is also depicted in Fig 1a. The proportion of TA-P < 3.4 mg/dL decreased while TA-P ≥ 3.5 mg/dL increased resulted from the progression of CKD. The relationship between baseline phosphorus and TA-P is plotted in Fig 1b with a correlation coefficient of 0.69 (*p* < 0.001). The regression line between TA-P versus baseline phosphorus was less than a unity in the slope and converged at 3.9 mg/dL of phosphorus due to the increase in serum phosphorus in the follow-up in the lower phosphorus groups.

### Standard Cox regression with time-averaged phosphorus

**Fig 2:**
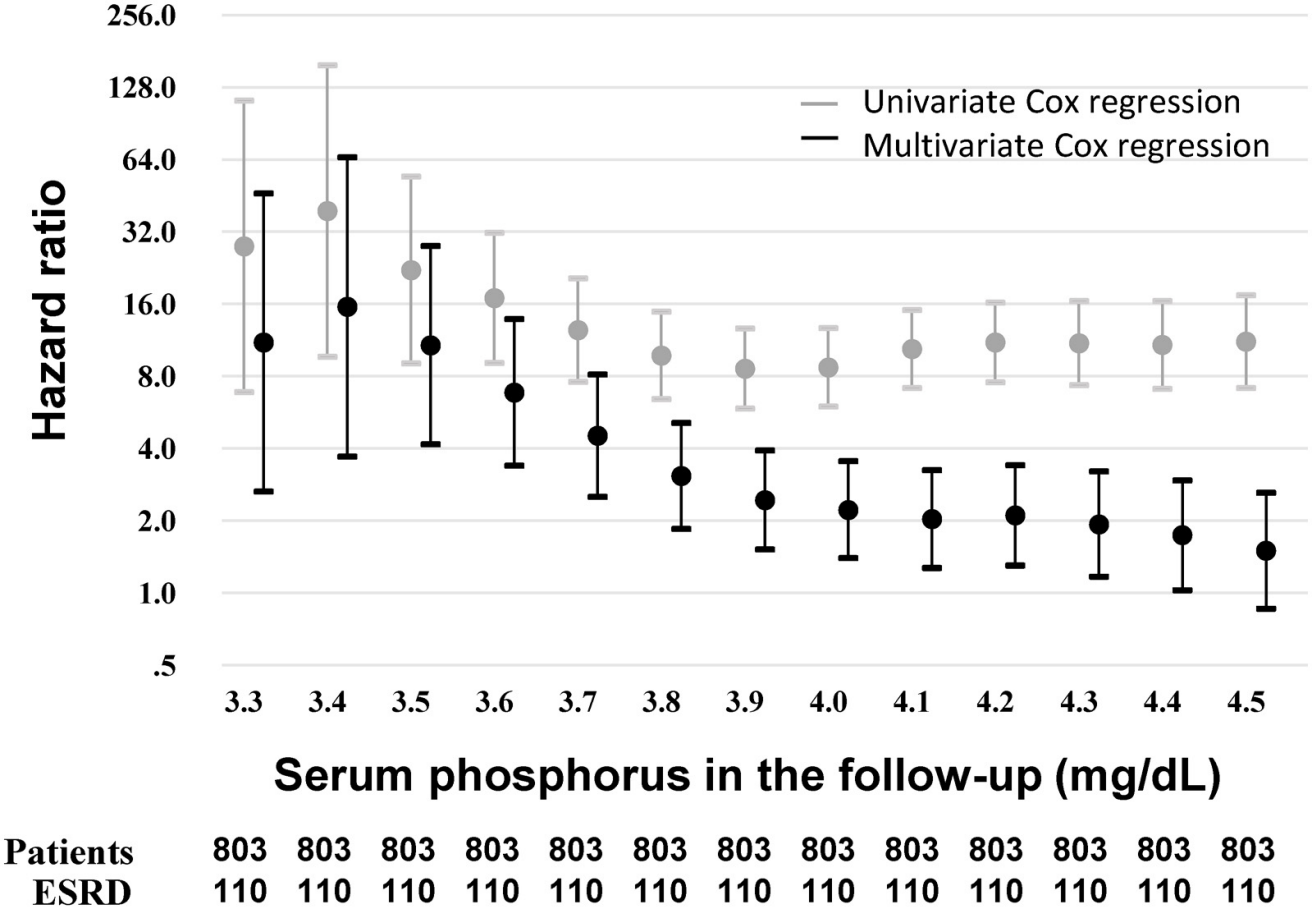
Hazard ratios at the different thresholds of serum phosphorus in the follow-up. Hazard ratios above versus below the indicated threshold of TA-P was obtained by applying standard Cox proportional hazards models. The total patients (n = 803) were separated at each threshold followed by survival analysis for ESRD as outcome (n = 110). Univariate analysis was performed for a threshold time-average phosphorus as a sole independent covariate. Multivariate analysis was adjusted for the baseline covariates including sex, age, diabetic nephropathy, estimated GFR, albumin, Na-Cl, phosphorus, LDL-C and proteinuria. The multivariate regression analysis showed the significant hazard ratio of higher TA-P up to 4.4 mg/dL. Abbreviation: TA-P, time-averaged phosphorus.

A time-to-event survival analysis was performed with TA-P for predicting the incidence of ESRD. TA-P was divided by a threshold from 3.3 to 4.5 mg/dL by a 0.1 mg/dL increment. A dichotomous value of TA-P thus obtained and other baseline covariates were tested for proportional hazards assumption, which turned out not violated. Multicollinearity was not observed, either. Crude HRs and its 95% confidence interval (CI) are plotted in Fig 2, and the HR was highest at the threshold of TA-P 3.4 mg/dL and gradually decreased but remained significantly as high a HR as approximately 10. Then, the multivariate Cox regression analysis adjusted for sex, age, diabetic nephropathy, estimated GFR, albumin, Na-Cl, phosphorus, LDL-C and proteinuria showed that the highest HR was also at the threshold of TA-P 3.4 mg/dL. Adjusted HR strikingly decreased greater than half the crude HR at any threshold of TA-P and lost its statistical significance at TA-P 4.5 mg/dL (Fig 2). However, these analyses *per se* could not provide target range of phosphorus to inhibit CKD progression. Also, the impacts of the time-varying parameter on CKD progression in the early stage and the late stage of the clinical course vary with time thus the time-averaged value is not appropriate for the risk factor analysis.

### A propensity score analysis

#### Stratified Cox proportional hazards model

A propensity score analysis could overcome the aforementioned issue. The threshold of TA-P was examined in an incremental way by 0.1 mg/dL from 3.3 to 4.5 mg/dL similar to the above. The accuracy and goodness-of-fit of the propensity score model are shown in S1 Table. C-statistics showed all greater than 0.8, indicating good accuracy of the model [22], whereas Hosmer-Lemeshow test for goodness-of-fit showed *p* < 0.05 in some situations (S1 Table). The effort to increase the *p* value > 0.05 by employing interaction terms and/or trimming more patients was not tried because Hosmer-Lemeshow test is not necessary recommended nowadays [23, 24].

**Fig 3:**
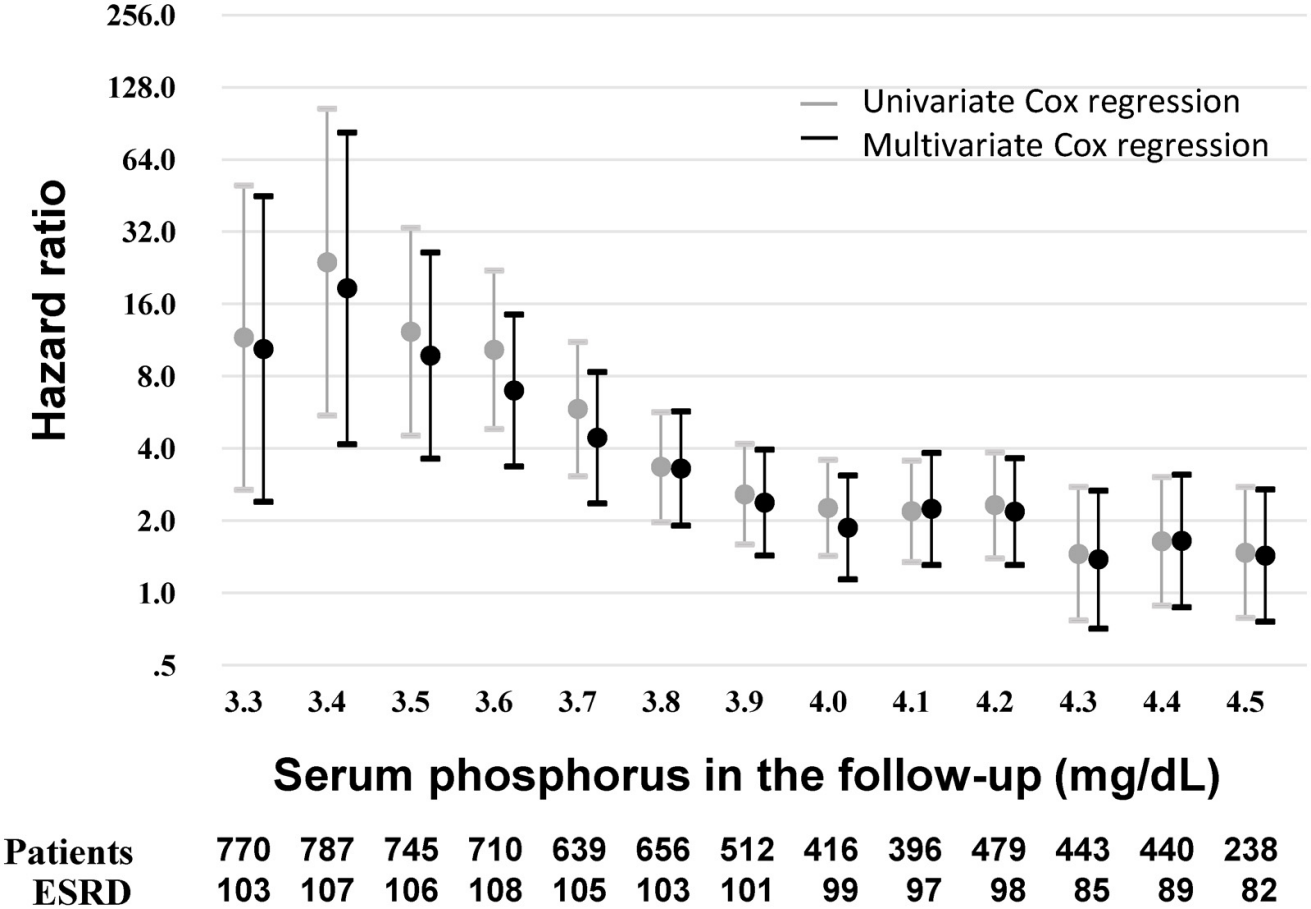
Hazard ratios at the different thresholds of serum phosphorus in the follow-up by propensity score analysis. Hazard ratios (HRs) below and above the indicated threshold of TA-P by applying stratified Cox proportional hazards models. The total patients (n = 803) were trimmed by removing the participants with non-overlapped propensity scores at each threshold. The numbers of test participants and ESRD are shown below the graph. Univariate analysis was performed for TA-P as a sole independent covariate. Multivariate analysis was adjusted for the baseline covariates including sex, age, diabetic nephropathy, estimated GFR, albumin, Na-Cl, phosphorus, LDL-C and proteinuria. The multivariate regression analysis showed the significant HR of higher TA-P up to 4.2 mg/dL. Abbreviation: TA-P, time-averaged phosphorus.

A subsample after trimming non-overlapped patients was re-stratified on the quintiles of the propensity scores, then subjected to survival analysis by a stratified multivariate Cox proportional hazards model. Proportional hazards assumption was not violated and multicollinearity was not seen. The result disclosed that the significantly high HRs, either crude or adjusted, were uniformly found from 3.3 to 4.2 mg/dL of TA-P (Fig 3) and that the HR was highest at the threshold of 3.4 mg/dL (crude HR 23.98, 95% CI 5.51–104.35; adjusted HR 18.60, 95% CI 4.17–82.98) and decreased thereafter but remained statistically significant up to 4.2 mg/dL (HR 2.33, 95% CI 1.40–3.86; adjusted HR 2.18, 95% CI 1.31–3.64). Of note is that the threshold at 4.3 mg/dL or higher lost the statistical significance with or without adjustment for other covariates (Fig 3). Moreover, Akaike information criterion significantly decreased (all *p* < 0.05; S1 Table) because a decrease in AIC ≥ 7 has been proposed to represent statistical significance at *p* value < 0.05 [25]. These results suggest that the stratified Cox proportional hazards model adjusted for multiple baseline covariates demonstrates the better goodness-of-fit.

Surprisingly the patterns of HRs of two different methods looked quite similar between the standard multivariate Cox regression models and the stratified multivariate Cox regression models upon propensity score analysis (Figs 2 and 3), suggesting that TA-P can be used for survival analysis probably due to the constant impact of serum phosphorus on kidney injury; in other words, no legacy effect is conceivable.

#### Propensity score matching

**Fig 4:**
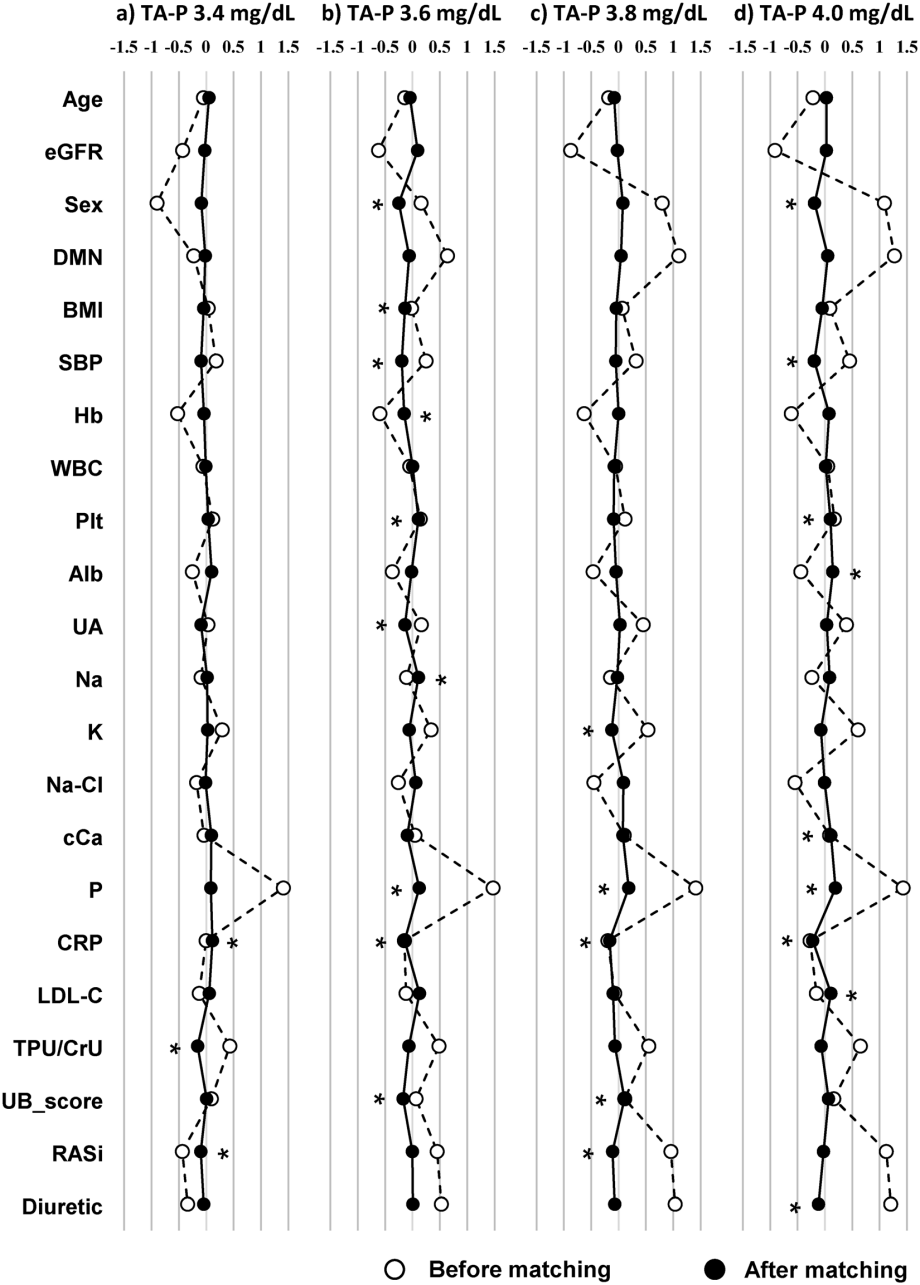
Standardized differences of baseline covariates before and after propensity score matching with different thresholds of serum phosphorus in the follow-up. **a)** 3.4 mg/dL, **b)** 3.6 mg/dL, **c)** 3.8 mg/dL, **d)** 4.0 mg/dL of serum phosphorus in the follow-up. Standardized differences below 0.1 are recognized as good balance between two groups. Covariates which did not reach below 0.1 is indicated by asterisk (*). Abbreviations: TA-P, time-averaged phosphorus; eGFR, estimated glomerular filtration rate; DMN, diabetic nephropathy; BMI, body mass index; SBP, systolic blood pressure; Hb, hemoglobin; WBC, white blood cell; Plt, platelet; Alb, albumin; UA, uric acid; Na, sodium; K, potassium; Cl, chloride; cCa, albumin-corrected calcium; P, phosphorus; CRP, C reactive protein; LDL-C, low-density lipoprotein cholesterol; TPU/CrU, urine total protein divided by urine creatinine; UB_score, urine blood score; RASi, RAS inhibitor.

**Fig 5:**
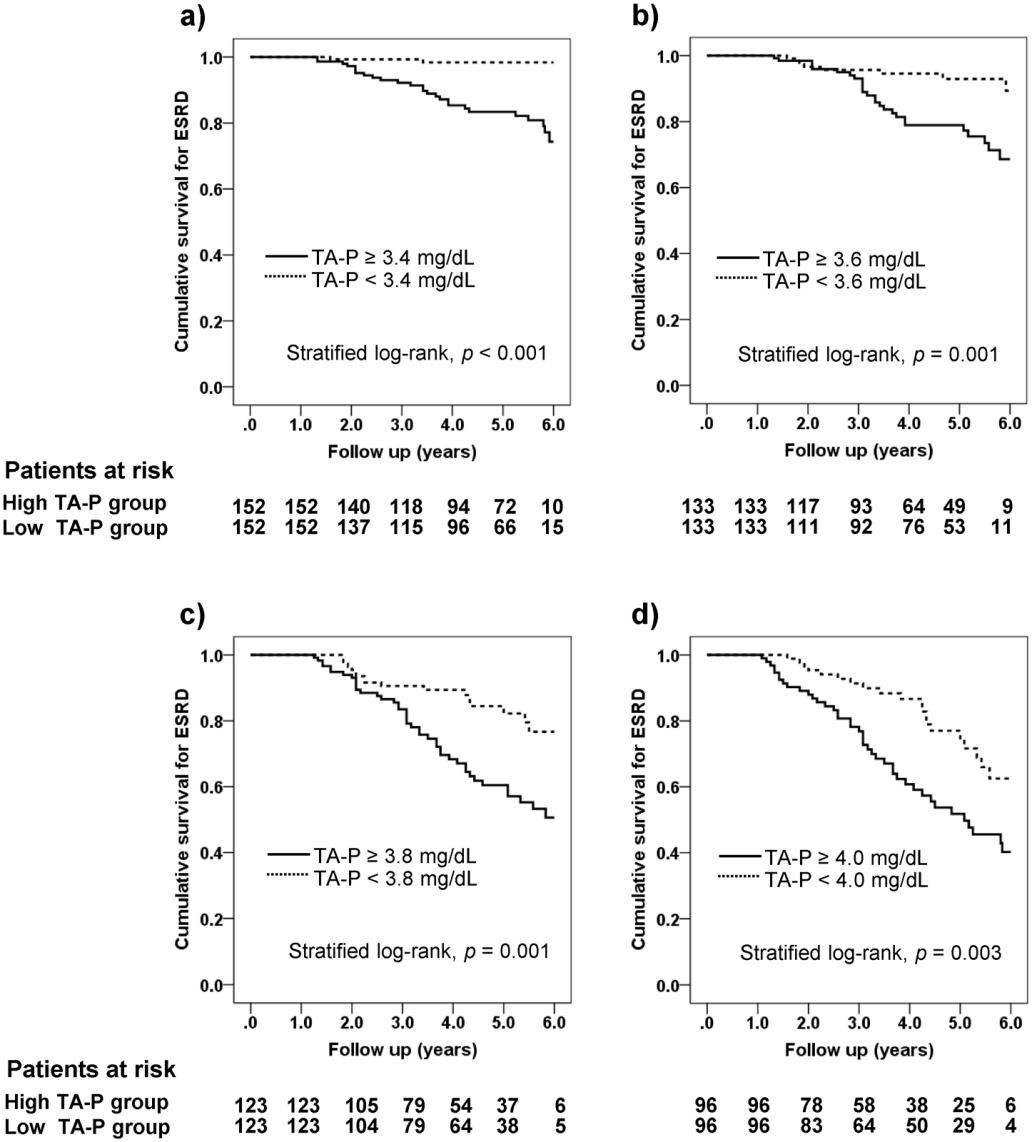
Kaplan-Meier plots separated by different thresholds of serum phosphorus in the follow-up after the propensity score matching. **a)** 3.4 mg/dL, **b)** 3.6 mg/dL, **c)** 3.8 mg/dL, **d)** 4.0 mg/dL of serum phosphorus in the follow-up. The risk tables are shown below the graph and *p* values are computed by stratified log-rank test. Hazard ratios of the incidence of ESRD regarding the thresholds of serum phosphorus in the follow-up were 12.83 (95% CI 6.12–21.92), 3.43 (95% CI 1.75–6.71), 2.56 (95% CI 1.54–4.26) and 2.29 (95% CI 1.39–3.79), respectively (See Table 2).

Propensity score matching was conducted with four different thresholds of 3.4, 3.6, 3.8 and 4.0 mg/dL of TA-P. Baseline covariates before and after matching were shown in S2–S5 Tables. Following matching, all the baseline covariates were well balanced not only by paired analyses (S2, S3, S4 and S5 Tables) but also by standardized differences (Fig 4). Some of the covariates, however, showed their standardized differences ≥ 0.1 as shown in Fig 4. The differences in mean TA-P between two groups after matching showed 0.5 to 0.7 mg/dL (S2, S2, S3, S4 and S5 Tables). Then, two groups divided by the threshold of TA-P were subjected to the Kaplan-Meier analysis (Table 2), the results of which are plotted in Fig 5a–5d. The patients with the higher TA-P showed significantly higher HRs for ESRD irrespective of the thresholds (3.4 mg/dL, HR 12.83, 95% CI 6.12–21.92; 3.6 mg/dL, HR 3.43, 95% CI 1.75–6.71; 3.8 mg/dL, HR 2.56, 95% CI 1.54–4.26; 4.0 mg/dL, HR 2.29, 95% CI 1.39–3.79; all *p* < 0.05 by stratified log-rank test). These results were consistent with those of the stratified multivariate Cox proportional hazards models (Fig 3). Numbers needed to treat in the four thresholds of TA-P were between 3.92 and 5.26 (Table 2), regarded as very small numbers [15, 26]. The result implies that targeting serum phosphorus to the lower normal range in 4 to 5 patients may save one extra patient from entering dialysis in the clinical course of CKD. Of another interest is that NNTs were very close despite the large differences in HRs among different thresholds, suggesting that CKD patients can receive the additional benefit whenever starting phosphorus-lowering treatment even before frank hyperphosphatemia occurs.

**Table 2:** Kaplan-Meier analysis before and after propensity score matching, and hazard ratio, absolute risk reduction and number needed to treat over 5 years of follow-up.

Threshold of TA-P	Before matching			After matching					
	Low (ESRD)	High (ESRD)	*p* value (log-rank)	Low (ESRD)	High (ESRD)	*p* value (stratified log-rank)	HR (95% CI)	ARR (95% CI)	NNT (95% CI)
≥ 3.4 mg/dL	277 (2)	446 (97)	< 0.001	152 (2)	152 (26)	< 0.001	12.83 (6.12–21.92)	0.25 (0.14–0.35)	4.08 (2.89–6.92)
≥ 3.6 mg/dL	443 (11)	280 (83)	< 0.001	133 (8)	133 (26)	0.001	3.43 (1.75–6.71)	0.24 (0.10–0.38)	4.14 (2.61–10.08)
≥ 3.8 mg/dL	558 (29)	165 (62)	< 0.001	123 (17)	123 (42)	0.001	2.56 (1.54–4.26)	0.26 (0.07–0.44)	3.92 (2.27–14.41)
≥ 4.0 mg/dL	625 (47)	98 (43)	< 0.001	96 (20)	96 (41)	0.003	2.29 (1.39–3.79)	0.19 (0.03–0.41)	5.26 (2.44–33.75)

Abbreviations: TA-P, time-averaged phosphorus; ESRD, end-stage renal disease; HR, hazard ratio; ARR, absolute risk reduction; NNT, Number needed to treat.

## Discussion

In the present study we could show the significant impact of the normal range of serum phosphorus in the follow-up on the subsequent incidence of ESRD by applying the propensity score analysis. Hazard ratios of the higher TA-P versus the lower group were uniformly high with the highest being at 3.4 mg/dL. Adjustment for other covariates assured the significance below 4.3 mg/dL. To our astonishment, the target value of phosphorus in the follow-up should be below 4.3 mg/dL and follows the theory of “the lower the better.” The present study is the first report elucidating that even the normal level of serum phosphorus in the follow-up underscores a risk factor of CKD progression, which was elucidated by applying the propensity score analysis.

A propensity score analysis has come into rapid use in the literature because it can approximate randomized controlled trials using retrospective observational cohorts. The method also enables one to investigate the causal effect which cannot be otherwise executed due to ethical issues. The advantage and disadvantage of the three approaches of the propensity score analysis established by Rosenbaum and Rubin [27] were discussed in more detail in the previous report [8]. In the present study, NNTs for targeting phosphorus in the follow-up were computed at 3.92 to 5.26 depending on the threshold, which indicates that we can rescue one extra patient if serum phosphorus is adequately treated in 4 to 5 patients over 5 years. The number was yet lower than that of the uric acid study [8], certainly encouraging clinicians to intervene serum phosphorus in the follow-up.

Since phosphorus and CKD constitutes a typical “chicken and egg problem,” one should be cautious about the cause and result relationship. Past studies indicated that hyperphosphatemia may play a pathogenic role in CKD progression [28]. Recently, meta-analysis of cohort studies (25,546 patients) indicates that every 1 mg/dL increase in serum phosphate level was associated independently with increased risk of kidney failure (HR 1.36; 95% CI 1.20–1.55) [29]. However, serum phosphorus was tested only once at baseline. Serum phosphorus tends to increase with an advancement of kidney dysfunction [30], regarded as one of the time-varying parameters [4, 5]. Thus, TA-P is better for assessing the impact of serum phosphorus on CKD progression. In practice, arithmetically calculated time-averaged values have been utilized in time-to-event survival analyses [31–34]. Most recently, the interesting study on ideal metrics of proteinuria in terms of risk factor analysis in patients with several types of glomerulonephritis was reported and concluded that the most ideal metric is time-varying method which uses the proteinuria value at every visit for time-varying Cox regression analysis [25]. A disadvantage of time-varying analysis is viewed not to yield a single value unlike baseline or time-averaged value, getting physicians embarrassed to grasp the clinical guide for treatment. In contrast, propensity score analysis by use of time-averaged values of test parameters has a great advantage to freely scrutinize the threshold of target values as demonstrated here and previously [8].

Worth mentioning is that no independent association of serum phosphorus with risk for CKD progression to ESRD was reported recently [10]. Mean phosphorus was even higher in their cohort as compared with our participants (3.74 vs. 3.37 mg/dL). One likely explanation for the contradiction may be ascribed to the better kidney function of their cohort (estimated GFR 48 vs. 41 mL/min/1.73 m^2^) and the shorter observation period (median 2.3 vs. 4.3 years) as compared with our cohort. Accordingly, an overall incidence rate of ESRD was 8.1 per 1,000 person-years much less than 33.9 in the present study. Therefore, the extended observation period of their study may be warranted.

The most striking finding in the present study is that such a low concentration of serum phosphorus as 3.4 mg/dl, well within the normal range, can be a risk factor of the progression of CKD to ESRD. The mechanism by which higher phosphorus can aggravate kidney injury may be partially extrapolated to this clinical scenario as suggested previously [35–37] but the specific pathophysiology should be considered in light of the particular setting of CKD because the normal range of serum phosphorus in healthy subjects never hurts kidneys. Firstly, FGF-23 is elevated before serum phosphorus is high and FGF-23 increases renal excretion of phosphate and maintains serum phosphate within the normal range but the increase in FGF-23 *per se* may exacerbate kidney dysfunction [36, 38]. Secondly, the increased phosphorus flux through the kidney tubules may accelerate the progression of CKD due to formation of calcium-phosphate crystals called calciprotein particles [38]. Thirdly, the uptake of phosphorus via Pit-1 protein has been shown to cause podocyte injuries in overexpression of Pit-1 in rats [39]. In any event the present finding may open up a new horizon for the impact of the normal range of serum phosphorus on the progression of CKD.

We have to mention about limitations of the present study. The first problem is the potential presence of unmeasured confounders which cannot be avoided in any observation study. Next, there is a possibility of misspecification of the propensity score model which cannot be asserted by any means, either. We believe the latter problem could be solved by accommodating a stratified multivariate Cox proportional hazards model as herein demonstrated. Lastly, this study was not a therapeutic trial and not designed to assess the impact of serum phosphorus targeting to lower normal range on the progression of CKD. Despite these limitations, a propensity score analysis clearly captures its unbounded potential to examine many test conditions such as risk factors and target thresholds. Needless to say, randomized controlled trials remain the gold standard to build evidence while a propensity score analysis may serve complementary approaches in the clinical research on the causal effect [8].

## Conclusion

The propensity score analysis unveiled that even normal levels of serum phosphorus in the follow-up can predict the risk of ESRD. Target range of serum phosphorus in the follow-up may be less than 4.3 mg/dL to inhibit the progression of CKD to ESRD with “the lower the better” principle. The results may facilitate vigorous treatment for lowering serum phosphorus level in clinical practice. The mechanism whereby normal range of serum phosphorus render kidney injury in the setting of CKD should be elucidated in animal and human studies.

## Supporting Information

S1 Table Evaluation of propensity score models and the goodness-of-fit of the result of stratified multivariate Cox regression. (DOCX)

S2 Table Covariates balance before and after matching divided by 3.4 mg/dL of time-averaged phosphorus in the follow-up. (DOCX)

S3 Table Covariates balance before and after matching divided by 3.6 mg/dL of time-averaged phosphorus in the follow-up. (DOCX)

S4 Table Covariates balance before and after matching divided by 3.8 mg/dL of time-averaged phosphorus in the follow-up. (DOCX)

S5 Table Covariates balance before and after matching divided by 4.0 mg/dL of time-averaged phosphorus in the follow-up. (DOCX)
