# Supplementary material for: The Impact of Normal Range of Serum Phosphorus on the Incidence of End-Stage Renal Disease by A Propensity Score Analysis
Source: PLoS One. 2016 Apr 28;11(4):e0154469. doi: 10.1371/journal.pone.0154469 (PMC4849666; doi:10.1371/journal.pone.0154469)
Supplement: S1 Table — (DOCX) [file pone.0154469.s001.docx]

**S1 Table. Evaluation of propensity score models and the goodness-of-fit of the result of stratified multivariate Cox regression**

| Threshold of TA-P | C-statistics of PS  AUROC curve | Hosmer-Lemeshow test  *p* value | Non-overlapped patients by PS (n =) | Stratified multivariate Cox regression | |
| --- | --- | --- | --- | --- | --- |
|  |  |  |  | AIC (Univariate) | AIC (Multivariate) |
| 3.0 mg/dL | 0.921 | 0.01 | 87 | 768 | 581 |
| 3.1 mg/dL | 0.899 | 0.01 | 96 | 671 | 520 |
| 3.2 mg/dL | 0.901 | 0.4 | 79 | 726 | 570 |
| 3.3 mg/dL | 0.871 | 0.02 | 33 | 894 | 722 |
| 3.4 mg/dL | 0.878 | 0.4 | 16 | 921 | 744 |
| 3.5 mg/dL | 0.884 | 0.2 | 58 | 896 | 717 |
| 3.6 mg/dL | 0.902 | 0.001 | 93 | 894 | 741 |
| 3.7 mg/dL | 0.908 | < 0.001 | 164 | 859 | 719 |
| 3.8 mg/dL | 0.915 | 0.01 | 147 | 859 | 722 |
| 3.9 mg/dL | 0.922 | 0.4 | 205 | 800 | 670 |
| 4.0 mg/dL | 0.919 | 0.5 | 387 | 744 | 636 |
| 4.1 mg/dL | 0.946 | 0.6 | 407 | 721 | 643 |
| 4.2 mg/dL | 0.948 | 0.2 | 324 | 764 | 689 |
| 4.3 mg/dL | 0.959 | 0.05 | 360 | 665 | 592 |
| 4.4 mg/dL | 0.967 | 0.002 | 363 | 695 | 619 |
| 4.5 mg/dL | 0.983 | < 0.001 | 565 | 538 | 479 |

Abbreviations: TA-P, time-averaged phosphorus; PS, propensity score; AUROC, area under the receiver operating characteristic; AIC, Akaike information criterion
