# Supplementary material for: The Impact of Normal Range of Serum Phosphorus on the Incidence of End-Stage Renal Disease by A Propensity Score Analysis
Source: PLoS One. 2016 Apr 28;11(4):e0154469. doi: 10.1371/journal.pone.0154469 (PMC4849666; doi:10.1371/journal.pone.0154469)
Supplement: S2 Table — (DOCX) [file pone.0154469.s002.docx]

**S2 Table. Covariates balance before and after matching divided by 3.4 mg/dL of time-averaged phosphorus in the follow-up**

| **Characteristics** | **Before matching (n = 803)** | | |  | **After matching (n =304)** | | |
| --- | --- | --- | --- | --- | --- | --- | --- |
|  | **TA-P < 3.4 n = 314** | **TA-P ≥ 3.4 n = 489** | ***p* value^*^** |  | **TA-P < 3.4 n = 152** | **TA-P ≥ 3.4 n = 152** | ***p* value^†^** |
| TA-P (mg/dL) | 3.1±0.3 | 3.8±0.4 | < 0.001 |  | 3.1±0.2 | 3.6±0.3 | < 0.001 |
| Age (y) | 62.6±13.0 | 61.9±13.1 | 0.5 |  | 61.8±13.0 | 62.4±12.8 | 0.7 |
| Baseline eGFR (mL/min/1.73 m^2^) | 44.4±10.9 | 39.0±14.0 | < 0.001 |  | 42.9±11.3 | 42.6±13.9 | 0.8 |
| Sex |  |  | < 0.001 |  |  |  | 0.6 |
| Male (%) | 232(73.9) | 269(55.0) |  |  | 100(65.8) | 95(62.5) |  |
| Female (%) | 82(26.1) | 220(45.0) |  |  | 52(34.2) | 57(37.5) |  |
| DMN (%) | 42(13.4) | 144(29.4) | < 0.001 |  | 32(21.1) | 30(19.7) | 0.9 |
| BMI (kg/m^2^) | 24.2±4.3 | 24.4±4.4 | 0.6 |  | 24.3±4.5 | 24.1±4.5 | 0.7 |
| SBP (mmHg) | 135.1±21.4 | 138.8±20.5 | 0.01 |  | 137.5±22.1 | 135.7±17.2 | 0.4 |
| Blood Parameters |  |  |  |  |  |  |  |
| Hb (g/dL) | 13.4±1.8 | 12.5±1.9 | < 0.001 |  | 13.1±1.8 | 13.0±1.8 | 0.7 |
| WBC (×10^2^/μL) | 66.3±22.5 | 65.0±20.7 | 0.4 |  | 63.7±20.4 | 63.6±21.2 | 0.9 |
| Plt (×10^4^/μL) | 21.5±6.4 | 22.3±7.0 | 0.1 |  | 21.0±5.9 | 21.2±6.3 | 0.8 |
| Alb (g/dL) | 4.0±0.4 | 3.9±0.5 | 0.001 |  | 4.0±0.5 | 4.0±0.4 | 0.4 |
| UA (mg/dL) | 6.5±1.4 | 6.5±1.5 | 0.7 |  | 6.5±1.5 | 6.4±1.4 | 0.4 |
| Na (mEq/L) | 140.9±2.5 | 140.6±2.8 | 0.2 |  | 140.8±2.8 | 140.8±2.6 | 0.9 |
| K (mEq/L) | 4.4±0.5 | 4.5±0.5 | < 0.001 |  | 4.4±0.5 | 4.4±0.5 | 0.8 |
| Na-Cl (mEq/L) | 35.7±2.3 | 35.2±2.6 | 0.02 |  | 35.6±2.3 | 35.6±2.3 | 0.9 |
| cCa (mg/dL) | 8.9±0.5 | 8.8±0.5 | 0.6 |  | 8.8±0.4 | 8.9±0.4 | 0.4 |
| P (mg/dL) | 3.0±0.4 | 3.6±0.4 | < 0.001 |  | 3.2±0.4 | 3.2±0.4 | 0.1 |
| CRP (mg/dL) | 0.09 [0.05-0.20] | 0.08 [0.04-0.20] | 0.9 |  | 0.08 [0.05-0.15] | 0.75 [0.04-0.20] | 0.3 |
| LDL-C (mg/dL) | 113.2±29.9 | 109.4±30.8 | 0.08 |  | 110.8±27.9 | 112.4±27.7 | 0.6 |
| Urine Parameters (spot) |  |  |  |  |  |  |  |
| TPU/CrU (g/g Cr) | 0.30 [0.15-0.69] | 0.51[0.20-1.50] | < 0.001 |  | 0.40 [0.20-1.01] | 0.35 [0.15-0.82] | 0.2 |
| UB_score | 0.00 [0.00-0.50] | 0.00 [0.00-1.00] | 0.2 |  | 0.00 [0.00-0.50] | 0.00 [0.00-0.50] | 0.9 |
| Drug use |  |  |  |  |  |  |  |
| RASi (%) | 172 (54.8) | 265 (54.2) | 0.9 |  | 86 (56.6) | 79 (52.0) | 0.5 |
| Diuretic (%) | 36 (11.5) | 92 (18.8) | 0.006 |  | 23 (15.1) | 17 (11.2) | 0.4 |

Note: Values for categorical variables are given as number (percentage); values for continuous variables are given as mean ± standard deviation or median [interquartile range]. For statistical analyses, CRP, TPU/CrU, UB_score were log-transformed. Conversion factors for units: creatinine in mg/dL to µmol/L, x 88.4; uric acid in mg/dL to µmol/L, x 59.48.

Abbreviations: TA-P, time-averaged phosphorus; eGFR, estimated glomerular filtration rate; DMN, diabetic nephropathy; BMI, body mass index; SBP, systolic blood pressure; Hb, hemoglobin; WBC, white blood cell; Plt, platelet; Alb, albumin; UA, uric acid; Na, sodium; K, potassium; Cl, chloride; cCa, albumin-corrected calcium; P, phosphorus; CRP, C reactive protein; LDL-C, low-density lipoprotein cholesterol; TPU/CrU, urine total protein divided by urine creatinine; UB_score, urine blood score; RASi, RAS inhibitor.

^*^ Unpaired *t* test or chi square test as appropriate.

^†^ Paired *t* test or McNemar test as appropriate.
