# Supplementary material for: The Impact of Normal Range of Serum Phosphorus on the Incidence of End-Stage Renal Disease by A Propensity Score Analysis
Source: PLoS One. 2016 Apr 28;11(4):e0154469. doi: 10.1371/journal.pone.0154469 (PMC4849666; doi:10.1371/journal.pone.0154469)
Supplement: S3 Table — (DOCX) [file pone.0154469.s003.docx]

**S3 Table. Covariates balance before and after matching divided by 3.6 mg/dL of time-averaged phosphorus in the follow-up**

| **Characteristics** | **Before matching (n = 803)** | | |  | **After matching (n = 266)** | | |
| --- | --- | --- | --- | --- | --- | --- | --- |
|  | **TA-P < 3.6 n = 487** | **TA-P ≥ 3.6 n = 316** | ***p* value^*^** |  | **TA-P < 3.6 n = 133** | **TA-P ≥ 3.6 n = 133** | ***p* value^†^** |
| TA-P (mg/dL) | 3.2±0.3 | 4.0±0.4 | < 0.001 |  | 3.4±0.2 | 3.9±0.3 | < 0.001 |
| Age (y) | 62.9±12.9 | 61.0±13.2 | 0.05 |  | 62.9±13.4 | 62.4±12.6 | 0.7 |
| Baseline eGFR (mL/min/1.73 m^2^) | 44.3±11.4 | 36.3±14.3 | < 0.001 |  | 39.2±11.1 | 40.4±14.0 | 0.5 |
| Sex |  |  | 0.003 |  |  |  | 0.2 |
| Male (%) | 324(66.5) | 177(56.0) |  |  | 87(65.4) | 74(55.6) |  |
| Female (%) | 163(33.5) | 139(44.0) |  |  | 46(34.6) | 59(44.4) |  |
| DMN (%) | 82(16.8) | 104(32.9) | < 0.001 |  | 38(28.6) | 32(24.1) | 0.5 |
| BMI (kg/m^2^) | 24.4±4.4 | 24.3±4.4 | 0.8 |  | 24.3±4.0 | 23.7±4.4 | 0.3 |
| SBP (mmHg) | 135.3±20.6 | 140.5±21.0 | 0.001 |  | 138.5±21.5 | 134.6±18.8 | 0.2 |
| Blood Parameters |  |  |  |  |  |  |  |
| Hb (g/dL) | 13.3±1.8 | 12.2±1.9 | < 0.001 |  | 12.7±1.8 | 12.4±1.9 | 0.2 |
| WBC (×10^2^/μL) | 65.9±21.9 | 64.9±20.7 | 0.5 |  | 64.5±18.6 | 64.5±23.0 | 0.9 |
| Plt (×10^4^/μL) | 21.6±6.6 | 22.6±7.1 | 0.05 |  | 21.4±6.8 | 22.2±7.7 | 0.4 |
| Alb (g/dL) | 4.0±0.4 | 3.8±0.5 | < 0.001 |  | 4.0±0.4 | 4.0±0.5 | 0.9 |
| UA (mg/dL) | 6.4±1.4 | 6.6±1.5 | 0.02 |  | 6.6±1.3 | 6.4±1.5 | 0.2 |
| Na (mEq/L) | 140.8±2.5 | 140.5±2.9 | 0.1 |  | 140.4±3.1 | 140.8±2.9 | 0.4 |
| K (mEq/L) | 4.4±0.5 | 4.6±0.6 | < 0.001 |  | 4.5±0.5 | 4.5±0.5 | 0.6 |
| Na-Cl (mEq/L) | 35.6±2.3 | 35.0±2.7 | < 0.001 |  | 35.3±2.9 | 35.4±2.5 | 0.6 |
| cCa (mg/dL) | 8.8±0.5 | 8.9±0.5 | 0.5 |  | 8.9±0.5 | 8.8±0.5 | 0.5 |
| P (mg/dL) | 3.1±0.4 | 3.8±0.4 | < 0.001 |  | 3.5±0.3 | 3.6±0.3 | 0.1 |
| CRP (mg/dL) | 0.09 [0.05-0.21] | 0.07 [0.04-0.17] | 0.05 |  | 0.10 [0.04-0.26] | 0.07 [0.05-0.20] | 0.2 |
| LDL-C (mg/dL) | 112.3±30.1 | 108.7±31.1 | 0.1 |  | 109.2±30.6 | 113.1±30.6 | 0.3 |
| Urine Parameters (spot) |  |  |  |  |  |  |  |
| TPU/CrU (g/g Cr) | 0.30 [0.15-0.78] | 0.82 [0.24-1.86] | < 0.001 |  | 0.40 [0.18-1.05] | 0.41 [0.16-1.14] | 0.6 |
| UB_score | 0.00 [0.00-0.50] | 0.50 [0.00-1.00] | 0.4 |  | 0.00 [0.00-1.00] | 0.00 [0.00-0.50] | 0.2 |
| Drug use |  |  |  |  |  |  |  |
| RASi (%) | 262 (53.8) | 175 (55.4) | 0.7 |  | 68 (51.1) | 68 (51.1) | 1.00 |
| Diuretic (%) | 61 (12.5) | 67 (21.2) | 0.002 |  | 20 (15.0) | 21 (15.8) | 1.00 |

Note: Values for categorical variables are given as number (percentage); values for continuous variables are given as mean ± standard deviation or median [interquartile range]. For statistical analyses, CRP, TPU/CrU, UB_score were log-transformed. Conversion factors for units: creatinine in mg/dL to µmol/L, x 88.4; uric acid in mg/dL to µmol/L, x 59.48.

Abbreviations: TA-P, time-averaged phosphorus; eGFR, estimated glomerular filtration rate; DMN, diabetic nephropathy; BMI, Body Mass Index; SBP, systolic blood pressure; Hb, hemoglobin; WBC, white blood cell; Plt, platelet; Alb, albumin; UA, uric acid; Na, sodium; K, potassium; Cl, chloride; cCa, albumin-corrected calcium; P, phosphorus; CRP, C reactive protein; LDL-C, low-density lipoprotein cholesterol; TPU/CrU, urine total protein divided by urine creatinine; UB_score, urine blood score; RASi, RAS inhibitor.

^*^ Unpaired *t* test or chi square test as appropriate.

^†^ Paired *t* test or McNemar test as appropriate.
