# Supplementary material for: The Impact of Normal Range of Serum Phosphorus on the Incidence of End-Stage Renal Disease by A Propensity Score Analysis
Source: PLoS One. 2016 Apr 28;11(4):e0154469. doi: 10.1371/journal.pone.0154469 (PMC4849666; doi:10.1371/journal.pone.0154469)
Supplement: S4 Table — (DOCX) [file pone.0154469.s004.docx]

**S4 Table. Covariates balance before and after matching by 3.8 mg/dL of time-averaged phosphorus in the follow-up**

| **Characteristics** | **Before matching (n = 803)** | | |  | **After matching (n =246)** | | |
| --- | --- | --- | --- | --- | --- | --- | --- |
|  | **TA-P < 3.8 n = 603** | **TA-P ≥ 3.8 n = 200** | ***p* value^*^** |  | **TA-P < 3.8 n = 123** | **TA-P ≥ 3.8 n = 123** | ***p* value^†^** |
| TA-P (mg/dL) | 3.3±0.3 | 4.2±0.4 | < 0.001 |  | 3.5±0.2 | 4.1±0.3 | < 0.001 |
| Age (y) | 62.8±12.8 | 60.4±13.6 | 0.02 |  | 61.9±13.6 | 60.8±13.6 | 0.5 |
| Baseline eGFR (mL/min/1.73 m^2^) | 43.9±11.7 | 32.7±13.9 | < 0.001 |  | 36.1±11.9 | 35.8±14.3 | 0.9 |
| Sex |  |  | 0.2 |  |  |  | 0.7 |
| Male (%) | 385(63.8) | 116(58.0) |  |  | 71(57.7) | 75(61.0) |  |
| Female (%) | 218(36.2) | 84(42.0) |  |  | 52(42.3) | 48(39.0) |  |
| DMN (%) | 114(18.9) | 72(36.0) | < 0.001 |  | 34(27.6) | 38(30.9) | 0.7 |
| BMI (kg/m^2^) | 24.3±4.3 | 24.5±4.6 | 0.4 |  | 24.4±5.0 | 24.2±4.5 | 0.8 |
| SBP (mmHg) | 135.6±20.1 | 142.4±22.5 | < 0.001 |  | 141.7±21.3 | 140.6±21.2 | 0.7 |
| Blood Parameters |  |  |  |  |  |  |  |
| Hb (g/dL) | 13.2±1.8 | 11.9±2.0 | < 0.001 |  | 12.3±1.8 | 12.3±2.1 | 0.9 |
| WBC (×10^2^/μL) | 65.8±21.8 | 64.6±20.4 | 0.5 |  | 67.4±20.3 | 65.6±22.0 | 0.5 |
| Plt (×10^4^/μL) | 21.8±7.0 | 22.6±63 | 0.2 |  | 22.7±7.4 | 22.1±6.0 | 0.5 |
| Alb (g/dL) | 4.0±0.5 | 3.8±0.5 | < 0.001 |  | 3.9±0.5 | 3.8±0.5 | 0.7 |
| UA (mg/dL) | 6.3±1.4 | 7.0±1.5 | < 0.001 |  | 6.8±1.4 | 6.9±1.5 | 0.8 |
| Na (mEq/L) | 140.8±2.6 | 140.4±2.8 | 0.06 |  | 140.8±2.3 | 140.7±2.7 | 0.9 |
| K (mEq/L) | 4.4±0.5 | 4.7±0.6 | < 0.001 |  | 4.6±0.5 | 4.5±0.6 | 0.3 |
| Na-Cl (mEq/L) | 35.7±2.3 | 345±2.8 | < 0.001 |  | 34.8±2.6 | 35.0±2.6 | 0.5 |
| cCa (mg/dL) | 8.8±0.5 | 8.9±0.5 | 0.2 |  | 8.9±0.4 | 8.9±0.5 | 0.5 |
| P (mg/dL) | 3.2±05 | 3.8±0.4 | < 0.001 |  | 3.6±0.4 | 3.7±0.4 | 0.06 |
| CRP (mg/dL) | 0.09 [0.05-0.20] | 0.07 [0.04-0.17] | 0.04 |  | 0.08 [0.04-0.20] | 0.08 [0.04-0.17] | 0.2 |
| LDL-C (mg/dL) | 111.4±30.3 | 109.3±31.2 | 0.4 |  | 112.0±31.4 | 108.9±31.7 | 0.4 |
| Urine Parameters (spot) |  |  |  |  |  |  |  |
| TPU/CrU (g/g Cr) | 0.33 [0.16-0.87] | 1.08 [0.41-2.35] | < 0.001 |  | 0.69 [0.25-2.32] | 0.87 [0.27-2.23] | 0.6 |
| UB_score | 0.00 [0.00-0.50] | 0.50 [0.00-1.00] | 0.2 |  | 0.00 [0.00-0.50] | 0.50 [0.00-1.00] | 0.4 |
| Drug use |  |  |  |  |  |  |  |
| RASi (%) | 322 (53.4) | 115 (57.5) | 0.3 |  | 72 (58.5) | 66 (53.7) | 0.5 |
| Diuretic (%) | 80 (13.3) | 48 (24.0) | 0.001 |  | 29 (23.6) | 22 (17.9) | 0.3 |

Note: Values for categorical variables are given as number (percentage); values for continuous variables are given as mean ± standard deviation or median [interquartile range]. For statistical analyses, CRP, TPU/CrU, UB_score were log-transformed. Conversion factors for units: creatinine in mg/dL to µmol/L, x 88.4; uric acid in mg/dL to µmol/L, x 59.48.

Abbreviations: TA-P, time-averaged phosphorus; eGFR, estimated glomerular filtration rate; DMN, diabetic nephropathy; BMI, Body Mass Index; SBP, systolic blood pressure; Hb, hemoglobin; WBC, white blood cell; Plt, platelet; Alb, albumin; UA, uric acid; Na, sodium; K, potassium; Cl, chloride; cCa, albumin-corrected calcium; P, phosphorus; CRP, C reactive protein; LDL-C, low-density lipoprotein cholesterol; TPU/CrU, urine total protein divided by urine creatinine; UB_score, urine blood score; RASi, RAS inhibitor.

^*^ Unpaired *t* test or chi square test as appropriate.

^†^ Paired *t* test or McNemar test as appropriate.
