# Supplementary material for: The Impact of Normal Range of Serum Phosphorus on the Incidence of End-Stage Renal Disease by A Propensity Score Analysis
Source: PLoS One. 2016 Apr 28;11(4):e0154469. doi: 10.1371/journal.pone.0154469 (PMC4849666; doi:10.1371/journal.pone.0154469)
Supplement: S5 Table — (DOCX) [file pone.0154469.s005.docx]

**S5 Table. Covariates balance before and after matching by4.0 mg/dL of time-averaged phosphorus in the follow-up**

| **Characteristics** | **Before matching (n = 803)** | | |  | **After matching (n =192)** | | |
| --- | --- | --- | --- | --- | --- | --- | --- |
|  | **TA-P < 4.0 n = 674** | **TA-P ≥ 4.0 n = 129** | ***p* value^*^** |  | **TA-P < 4.0 n = 96** | **TA-P ≥ 4.0 n = 96** | ***p* value^†^** |
| TA-P (mg/dL) | 3.4±0.4 | 4.3±0.4 | < 0.001 |  | 3.6±0.3 | 4.3±0.3 | < 0.001 |
| Age (y) | 62.6±13.0 | 59.8±13.4 | 0.02 |  | 59.5±14.2 | 59.9±12.9 | 0.8 |
| Baseline eGFR (mL/min/1.73 m^2^) | 43.0±12.0 | 31.0±14.3 | < 0.001 |  | 34.0±11.4 | 34.4±14.8 | 0.8 |
| Sex |  |  | 0.5 |  |  |  | 0.4 |
| Male (%) | 424(62.9) | 77(59.7) |  |  | 62(64.6) | 55(57.3) |  |
| Female (%) | 250(37.1) | 52(40.3) |  |  | 34(35.4) | 41(42.7) |  |
| DMN (%) | 137(20.3) | 49(38.0) | < 0.001 |  | 36(37.5) | 39(40.6) | 0.8 |
| BMI (kg/m^2^) | 24.3±4.3 | 24.7±4.6 | 0.3 |  | 25.0±4.6 | 24.7±4.5 | 0.7 |
| SBP (mmHg) | 135.8±20.4 | 145.4±21.9 | < 0.001 |  | 148.6±22.8 | 144.4±21.3 | 0.2 |
| Blood Parameters |  |  |  |  |  |  |  |
| Hb (g/dL) | 13.0±1.9 | 11.9±2.0 | < 0.001 |  | 12.1±2.0 | 12.2±1.9 | 0.6 |
| WBC (×10^2^/μL) | 65.3±21.6 | 66.4±20.5 | 0.6 |  | 66.6±18.2 | 66.9±21.4 | 0.9 |
| Plt (×10^4^/μL) | 21.8±6.8 | 23.0±6.7 | 0.07 |  | 22.3±6.9 | 23.0±6.4 | 0.5 |
| Alb (g/dL) | 4.0±0.5 | 3.8±0.5 | < 0.001 |  | 3.7±0.5 | 3.8±0.5 | 0.3 |
| UA (mg/dL) | 6.4±1.4 | 7.0±1.6 | < 0.001 |  | 6.8±1.4 | 6.9±1.5 | 0.8 |
| Na (mEq/L) | 140.8±2.7 | 140.2±2.7 | 0.01 |  | 140.2±2.9 | 140.5±2.5 | 0.6 |
| K (mEq/L) | 4.4±0.5 | 4.8±0.6 | < 0.001 |  | 4.7±0.5 | 4.6±0.6 | 0.6 |
| Na-Cl (mEq/L) | 35.6±2.4 | 34.2±2.9 | < 0.001 |  | 34.8±2.7 | 34.7±2.5 | 0.9 |
| cCa (mg/dL) | 8.8±0.5 | 8.9±0.5 | 0.4 |  | 8.8±0.5 | 8.9±0.5 | 0.4 |
| P (mg/dL) | 3.3±0.5 | 3.9±0.4 | < 0.001 |  | 3.7±0.4 | 3.8±0.3 | 0.1 |
| CRP (mg/dL) | 0.08 [0.05-0.20] | 0.08 [0.04-0.15] | 0.03 |  | 0.07 [0.04-0.17] | 0.08 [0.03-0.16] | 0.1 |
| LDL-C (mg/dL) | 111.6±30.4 | 106.9±30.8 | 0.1 |  | 106.4±31.8 | 109.9±31.1 | 0.5 |
| Urine Parameters (spot) |  |  |  |  |  |  |  |
| TPU/CrU (g/g Cr) | 0.36 [0.17-0.98] | 1.32 [0.48-2.52] | < 0.001 |  | 1.14 [0.34-2.51] | 1.16 [0.40-2.50] | 0.7 |
| UB_score | 0.00 [0.00-0.50] | 0.50 [0.00-1.00] | 0.09 |  | 0.50 [0.00-1.00] | 0.50 [0.00-1.00] | 0.6 |
| Drug use |  |  |  |  |  |  |  |
| RASi (%) | 367 (54.5) | 702 (54.3%) | 1.00 |  | 55 (57.3) | 54 (56.3) | 1.00 |
| Diuretic (%) | 98 (14.5) | 30 (23.3%) | 0.02 |  | 30 (31.3) | 22 (22.9) | 0.3 |

Note: Values for categorical variables are given as number (percentage); values for continuous variables are given as mean ± standard deviation or median [interquartile range]. For statistical analyses, CRP, TPU/CrU, UB_score were log-transformed. Conversion factors for units: creatinine in mg/dL to µmol/L, x 88.4; uric acid in mg/dL to µmol/L, x 59.48.

Abbreviations: TA-P, time-averaged phosphorus; eGFR, estimated glomerular filtration rate; DMN, diabetic nephropathy; BMI, Body Mass Index; SBP, systolic blood pressure; Hb, hemoglobin; WBC, white blood cell; Plt, platelet; Alb, albumin; UA, uric acid; Na, sodium; K, potassium; Cl, chloride; cCa, albumin-corrected calcium; P, phosphorus; CRP, C reactive protein; LDL-C, low-density lipoprotein cholesterol; TPU/CrU, urine total protein divided by urine creatinine; UB_score, urine blood score; RASi, RAS inhibitor.

^*^ Unpaired *t* test or chi square test as appropriate.

^†^ Paired *t* test or McNemar test as appropriate.
